# Unusual Sporotrichosis: A New Concept Proposal on the Unexpected Faces of *Sporothrix* spp. Infection

**DOI:** 10.3390/jof12020155

**Published:** 2026-02-21

**Authors:** Jayne Araújo da Silva, Adriany Lucas dos Santos, Júlia Andrade de Castro Rodrigues, Mariana de Paula Pires, Marcelo Cerilo-Filho, Gil Benard, José Rodrigo Santos Silva, Ricardo Luiz Dantas Machado, Jéssica Dornelas da Silva, Héctor Manuel Mora-Montes, Gutemberg Gomes Alves, Andréa Regina de Souza Baptista

**Affiliations:** 1 Graduate Program in Applied Microbiology and Parasitology, Biomedical Institute, Fluminense Federal University, Niteroi 24020-150, RJ, Brazil; jaynearaujo@id.uff.br (J.A.d.S.); adriany_lucas@id.uff.br (A.L.d.S.); mcerilo@id.uff.br (M.C.-F.); ricardomachado@id.uff.br (R.L.D.M.); 2 Center for Microorganisms’ Investigation, Department of Microbiology and Parasitology, Biomedical Institute, Fluminense Federal University, Niteroi 24020-141, RJ, Brazil; julia_andrade@id.uff.br (J.A.d.C.R.); piresmariana@id.uff.br (M.d.P.P.); 3 Laboratory of Medical Mycology (LIM-53), Division of Clinical Dermatology, Institute of Tropical Medicine, Hospital das Clínicas, Faculty of Medicine, Universidade de São Paulo, São Paulo 05403-000, SP, Brazil; bengil60@gmail.com; 4 Graduate Program in Biology of Infectious and Parasitic Agents, Centre for Biological and Health Sciences, Federal University of Sergipe, São Cristóvão 49100-000, SE, Brazil; rodrigo.silva@academico.ufs.br; 5 Clinical Research Unit of the Antonio Pedro Hospital, NanoOnco 3D, Fluminense Federal University, Niteroi 24033-900, RJ, Brazil; nanoonco3d@gmail.com; 6 Departamento de Biología, División de Ciencias Naturales y Exactas, Campus Guanajuato, Universidad de Guanajuato, Noria Alta s/n, col. Noria Alta, Guanajuato 36050, Mexico; hmora@ugto.mx; 7 Cell and Molecular Biology Department, Institute of Biology, Fluminense Federal University, Niteroi 24210-201, RJ, Brazil

**Keywords:** fungal infections, mycoses, unusual presentations, human health, systematic review

## Abstract

“Unusual sporotrichosis”, a concept proposed in this review, refers to severe, extracutaneous, or anatomically atypical manifestations of sporotrichosis occurring in immunocompetent hosts and represents an underrecognized clinical subset associated with important diagnostic and therapeutic challenges. This systematic review aimed to characterize unusual sporotrichosis worldwide and to clarify its epidemiological, clinical, diagnostic, and therapeutic patterns. Following a registered protocol and PRISMA guidelines, PubMed, Scopus, and BVS/LILACS were searched up to November 2025 using a PICO-based strategy. Eligible studies included peer-reviewed case reports and case series with laboratory-confirmed sporotrichosis in patients without immunosuppression, diabetes mellitus, alcoholism, or other confounding comorbidities; classical lymphocutaneous and fixed cutaneous forms were excluded. From 922 records, 39 studies were included (13 case series and 26 case reports), yielding 55 cases reported between 1957 and 2024 across five world regions, mainly from the United States of America and Brazil. Adults aged 40–59 years (41.8%) and males (74.5%) predominated. Sapronotic transmission was most frequent (69.0%), although zoonotic transmission increased over time. *Sporothrix schenckii/Sporothrix schenckii sensu stricto* was the predominant species (87.3%). Osteoarticular (30.9%) and systemic (27.2%) forms were the most common presentations. Although cure was achieved in most cases (58.1%), sequelae were frequent (21.8%), and the worst prognosis—including most deaths—was observed in osteoarticular sporotrichosis. Unusual sporotrichosis is globally distributed and clinically distinct; therefore, early recognition and multimodal diagnostic and therapeutic strategies are essential to improve outcome.

## 1. Introduction

Sporotrichosis is a subcutaneous mycosis with a global distribution, primarily occurring in tropical and subtropical regions, though isolated cases have also been documented in temperate climates [1]. The disease results from infection with pathogenic dimorphic fungi belonging to the genus *Sporothrix* (*Ophiostomatales*) [2]. *Sporothrix schenckii* was designated by Hektoen and Perkins following Benjamin Schenck’s identification of the infectious etiological agent of sporotrichosis in 1898 [2,3].

For an extended period, *Sporothrix schenckii* was considered the sole causative agent of the disease. Subsequent molecular research has identified genetic diversity among various fungal strains, revealing that this organism is a member of a species complex [2,4,5]. The *Sporothrix* species complex includes four principal species recognized as pathogenic to humans and other mammals: *Sporothrix brasiliensis*, *Sporothrix schenckii sensu stricto*, *Sporothrix globosa*, and *Sporothrix luriei* [4,6]. Geographical distribution, virulence and antifungal drug susceptibility differences among these species were reported [7].

The classical mode of infection occurs via the saprophytic route, wherein *Sporothrix* spp. conidia enter the host either through the respiratory tract or, more commonly, by traumatic inoculation into the subcutaneous tissue via contaminated organic matter. This traditional pathway is frequently associated with recreational and occupational activities such as floristry, gardening, farming, and mining [8]. Since the mid-1990s, and with increasing intensity in recent years, the disease has exhibited a marked shift toward epidemic and, over the past three decades, hyperendemic patterns in Brazil. This trend is primarily attributable to zoonotic transmission, most notably through bites and scratches inflicted by infected cats [9]. Among animal reservoirs, cats demonstrate the highest susceptibility to infection, and their lesions typically have a high fungal burden, making them particularly effective at transmitting the pathogen to humans and other mammals [7,9].

Human sporotrichosis shows a wide spectrum of clinical manifestations, encompassing both cutaneous and extracutaneous forms. The cutaneous forms are generally classified into three main categories: lymphocutaneous, fixed cutaneous, and disseminated cutaneous types. The lymphocutaneous form, the most frequent, is distinguished by the development of erythematous nodules that progress along the lymphatic drainage pathway originating from the site of inoculation [10]. The fixed cutaneous form is characterized by a solitary lesion at the inoculation site, which may present as papules, ulcers, or crusts [11]. The disseminated cutaneous form, which occurs less commonly, is characterized by multiple lesions distributed across various body regions without adherence to a lymphatic pattern [12]. Sporotrichosis can also affect extracutaneous sites, including the osteoarticular and central nervous systems, mucosa and lungs [12]. The pulmonary manifestation of sporotrichosis, while rare, results from the inhalation of fungal elements and has the potential to progress to systemic dissemination [13].

Key determinants of host–pathogen interaction ultimately define human sporotrichosis clinical presentations [14]. Disseminated cutaneous and extracutaneous sporotrichosis often occurs due to factors like lifestyle choices, existing medical conditions, or system dysfunctions. People who have HIV/AIDS, chronic alcoholism, diabetes mellitus, steroids use, anti-TNF treatment, hematologic cancer and transplanted patients are especially likely to develop these severe forms of the disease [12,14,15,16].

However, several reports indicate that severe *Sporothrix* spp. infectious manifestations are no longer confined to the described risk groups [17,18,19,20,21,22,23,24,25,26,27,28,29,30,31,32,33,34,35,36,37,38,39,40,41,42,43,44,45,46,47,48,49,50,51,52,53,54,55,56], underscoring the need for comprehensive clinical and epidemiological characterization of such presentations. Moreover, when human sporotrichosis presents unexpectedly, misdiagnosis is common, as these forms may mimic other infectious or inflammatory conditions, frequently resulting in appropriate treatment delay. Such cases often require prolonged antifungal therapy and are associated with higher rates of therapeutic failure, relapse, and increased morbidity and mortality. These observations suggest that sporotrichosis may present with severe or “atypical” manifestations even in the absence of classical host-related risk factors, indicating the emergence of a distinct clinical subset. In this context, this systematic review aims to systematically characterize these presentations and to propose the concept of ‘unusual sporotrichosis’ based on predefined clinical and host-related criteria.

Findings from this systematic review indicate that, despite the growing number of human sporotrichosis case reports, describing a variety of unusual clinical manifestations, the available literature remains fragmented, and key questions persist regarding the true prevalence of these forms, their potential relationship with specific *Sporothrix* species, and their clinical outcomes. Addressing these gaps through the systematic synthesis of global evidence is crucial for identifying novel susceptibility traits and transmission routes, refining diagnostic and therapeutic strategies, and strengthening public health responses, ultimately improving patient management and reducing the global impact of sporotrichosis.

## 2. Materials and Methods

### 2.1. Protocol and Registry

This systematic review was conducted according to the protocol registered in the Open Science Framework database, available at the link: https://archive.org/details/osf-registrations-q6uwb-v1 (accessed on 21 December 2025; DOI 10.17605/OSF.IO/Q6UWB), and reported following the Preferred Reporting Items for Systematic Reviews and Meta-analyses (PRISMA) statement [57]. The completed PRISMA checklist is provided as Supplementary File S1.

### 2.2. Information Sources and Search Strategy

The search strategy was developed using the PICO framework, focusing on these components: Population—human patients diagnosed with sporotrichosis; Intervention—unexpected or unusual cases without related immunodeficiencies, comorbidities, or alcoholism; Comparison—disease involvement and clinical form (single or multiple, cutaneous or extracutaneous), as well as etiological agent/species and epidemiology (such as infection source, prevalence, geographic distribution, and socio-demographic characteristics). Outcome measures included describing clinical cases with unusual forms of sporotrichosis, therapeutic regimens used, and patient outcomes (recovery, relapse, complications, or death).

The literature search was conducted up to November 2025 in PubMed, Scopus, and the Virtual Health Library (BVS—Biblioteca Virtual em Saúde), where the LILACS (Latin American and Caribbean Health Sciences Literature) filter was applied to capture regional scientific publications. In PubMed, the search was: (“Sporotrichosis” [MeSH] OR “Sporothrix Infection” OR “Sporothrix schenckii Infection” OR “Sporothrix brasiliensis Infection”) AND (“complication*” OR “unusual” OR “uncommon” OR “atypical”) AND (“human*”). For Scopus, the query used was: (“Sporotrichosis” OR “Sporotrichoses” OR “Sporothrix Infection” OR “Sporothrix Infections” OR “Sporothrix schenckii Infection” OR “Sporothrix schenckii Infections” OR “Sporothrix brasiliensis Infection” OR “Sporothrix brasiliensis Infections”) AND (“complication*” OR “unusual” OR “uncommon” OR “atypical”) AND (“human*”). In BVS, the search strategy included the following terms: (“Sporotrichosis” OR “Sporotrichoses” OR “Sporothrix Infection” OR “Sporothrix Infections” OR “Sporothrix schenckii Infection” OR “Sporothrix schenckii Infections” OR “Sporothrix brasiliensis Infection” OR “Sporothrix brasiliensis Infections”) AND (“complication*” OR “unusual” OR “uncommon” OR “atypical”) AND (“human*”). In all databases, search terms were used to target words in the title, abstract, and subject fields. The records were imported into Zotero software (version 7.0.24) for duplicate removal, and the final dataset was organized and managed in Microsoft Excel (Microsoft 365 Apps, version 2512) for analysis. In addition, during full-text assessment, studies that strictly fulfilled the operational definition of “unusual sporotrichosis” but were not fully captured by the initial search filters were eligible for inclusion based on thematic relevance.

### 2.3. Study Selection

In this review, “unusual sporotrichosis” refers to a predefined clinical category encompassing sporotrichosis cases that deviate from the classical lymphocutaneous or fixed cutaneous patterns from the severe forms in the previously recognized susceptible patients. This term is used as a concept-driven and operational category, intended to complement, rather than replace existing clinical classifications, and to reduce interpretative ambiguity during study selection and data analysis. This category includes extracutaneous, disseminated cutaneous, or anatomically atypical presentations occurring in immunocompetent hosts, in the absence of recognized predisposing conditions (e.g., diabetes mellitus, alcoholism, HIV infection, immunosuppressive therapy. Key clinical and operational terms used throughout this review are defined in the Glossary (Appendix C). This operational definition guided study selection, data extraction, and subsequent analyses.

The screening process was conducted in two stages: an initial review of titles and abstracts, when three reviewers independently screened the titles and abstracts retrieved through the search, following a calibration exercise that yielded a Cohen’s kappa coefficient of 0.97, indicating near-perfect agreement. In the second stage, full-text analysis was performed on the articles that met the initial eligibility criteria. Any discrepancies regarding study inclusion were resolved through discussion and consensus; when needed, a fourth reviewer was consulted to make the final decision.

Studies were selected based on eligibility criteria that focused on reports of unexpected cases or unusual case series of human sporotrichosis, following the PICO guidelines (Section 2.2). An unexpected/unusual case was defined as a rare form of the disease, confirmed both clinically and by laboratory tests in patients who had no immune deficiencies, predisposing comorbidities, such as diabetes mellitus, corticosteroid use, or history of alcoholism. Studies were included based on the following criteria: publication in peer-reviewed journals with detailed descriptions of sampling strategy and study design; use of laboratory diagnostic methods, such as *Sporothrix* spp. isolation in mycological culture, to confirm diagnosis; and the availability of demographic data on study populations, including age groups (children and/or adults) and regions or country of residence. Studies were excluded if the full text was unavailable or if an abstract was not provided. No restrictions were placed on the publication date, while there was a limit in languages (English, Portuguese, French, and Spanish), covering the period from the first reported case up to November 2025, without any time restrictions.

To ensure a focused and representative dataset, strict exclusion criteria were ap-plied. Non-human studies, including experimental animal models and in vitro research, were excluded. Studies involving forms of sporotrichosis that were cutaneous, extracutaneous, or disseminated, such as pulmonary or widespread cutaneous presentations, were also excluded when reported in high-risk populations such as individuals with alcoholism, diabetes, advanced immunosuppression (e.g., solid organ transplant recipients or those undergoing immunomodulatory therapy), and people living with HIV/AIDS. Furthermore, cases involving *Sporothrix* inoculation at unusual anatomical sites, as well as those presenting comorbidities that could confound clinical interpretation, were excluded. Additionally, records referring to publications with no accessible full text (e.g., abstract-only publications or sources without retrievable full-text versions) were excluded at the screening stage. Articles lacking relevant clinical comparisons or meaningful insights were excluded.

### 2.4. Critical Appraisal

The risk of bias in each study was independently assessed by two reviewers using the Joanna Briggs Institute (JBI) Critical Appraisal tools: the Case Report Checklist was applied to case reports [58], and the Case Series Checklist was used for case series [59]. The percentage of checklist items fulfilled was used to provide an overview of reporting completeness and potential sources of bias, rather than as a quantitative measure of study quality. The individual results for each study are reported on Appendix A. Formal assessment of reporting bias was not performed due to the nature of the evidence base, which consisted exclusively of case reports and case series.

### 2.5. Data Extraction

Data extraction focused on a comprehensive set of variables, including author(s) and year of publication, study type, world region, country, species, year of patient inclusion, age, sex, occupational or recreational exposure, diagnostic and screening methods, clinical presentation, treatment, and outcomes. Given the descriptive nature of the available evidence, outcomes of interest were defined as clinical presentation, anatomical involvement, severity markers, treatment, and reported clinical outcomes. Primary outcomes included clinical outcomes (cure, improvement, sequelae, or death), while secondary outcomes comprised clinical form distribution, species identification, transmission route, and treatment patterns. The number of patients reported and those included in the study. Species-level classification was based on the diagnostic methods reported in each study and therefore reflects the technologies available at the time of publication, with molecular identification restricted to more recent cases. These collected data provided the foundation for a qualitative evaluation and synthesis of the evidence. Data extraction was performed independently by two reviewers using a standardized data collection form, with discrepancies resolved by consensus.

### 2.6. Data Analysis

All data from the eligible case reports and case series were first entered into a standard spreadsheet in Microsoft Excel (version 2024) for initial organization and consistency checking. The final database was then exported to R software (version 4.5.1) for analysis. Categorical variables (such as world region, sex, age group, route of transmission, clinical form, species, diagnostic method, treatment, and outcome) were described using percentages. Effect measures were expressed as proportions and differences in proportions between subgroups. Age was also summarized using simple descriptive measures. Exploratory subgroup analyses were conducted, and differences in proportions between groups were tested using Fisher’s Exact test. These tests were also used to compare how clinical forms varied by world region, age group, sex, time, and outcome. Given the descriptive nature of the evidence base and the heterogeneity of study designs, no formal meta-analysis of pooled effect measures was attempted, and these analyses were interpreted as hypothesis-generating rather than confirmatory. The level of statistical significance adopted for all tests was 5% (*p* < 0.05). No formal assessment of certainty of evidence was conducted, as the review was based on descriptive evidence from case reports and case series.

## 3. Results

**Figure 1:**
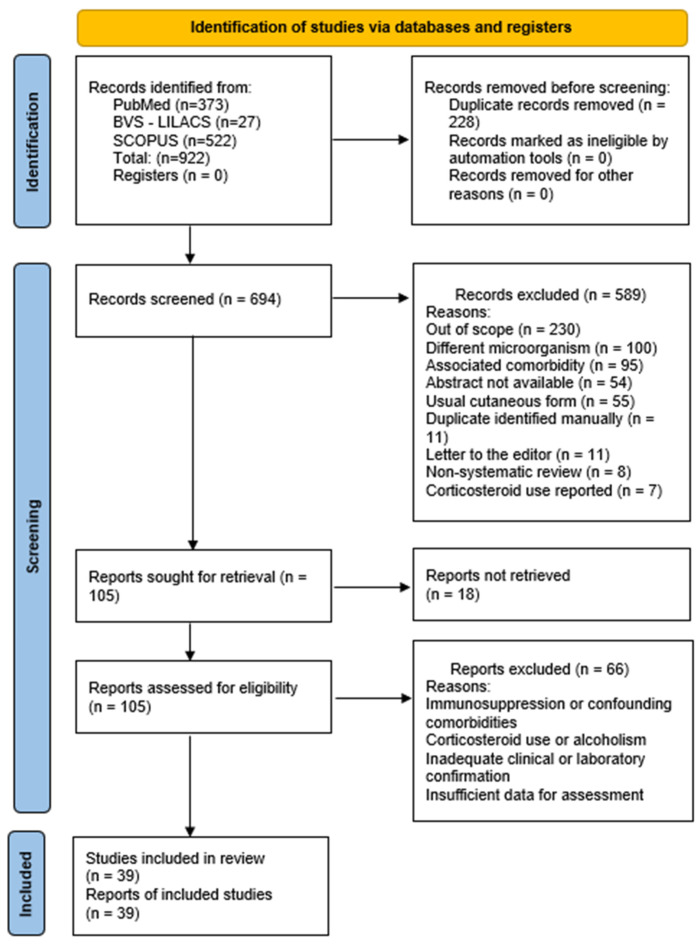
Flowchart of the screening process, selection, and systematic steps according to the PRISMA Statement 2020.

In the present review, a total of 922 records were identified. After removing 228 duplicates, 694 unique records remained. Screening of titles and abstracts resulted in the exclusion of 589 articles, as detailed in Figure 1. Full texts of 105 articles were then assessed, with 66 studies excluded for similar reasons, identified only upon full-text review. Of the remaining articles, 39 articles met the predefined inclusion criteria, totalling 39 studies in this review. Among the 39 included articles, 13 (33.3%) were case series, while 26 (66.7%) were case reports, yielding a total of 55 cases of unexpected sporotrichosis, according to the predefined operational definition employed in this review.

### 3.1. Critical Appraisal

The critical appraisal of case series revealed that their methodological quality varied from 50% to 88%, with most studies exhibiting problems in providing information about clinical settings and the sequential and complete inclusion of individuals (Table A1—Appendix A). Notably, the oldest studies presented moderate methodological quality (50%), whereas all subsequent studies scored above 60%, thus reflecting moderate to high quality with a lower risk of bias. Case reports generally achieved higher scores, with most of them fulfilling 81–100% of the requirements, reflecting clearer reporting of demographics, clinical course, diagnostic methods, and practical lessons, although adverse events were occasionally underreported. While case series offered more comprehensive evidence, they also carried a larger risk of bias. In general, case reports demonstrated superior methodological consistency.

### 3.2. Study Characteristics

Table 1 shows the demographic information from the selected studies [18,19,20,21,22,23,24,25,26,27,28,29,30,31,32,33,34,35,36,37,38,39,40,41,42,43,44,45,46,47,48,49,50,51,52,53,54,55,56,57]. Among the 39 included articles, 13 (33.3%) were case series and 26 (66.7%) were case reports, yielding a total of 55 cases. The 55 unusual cases of sporotrichosis were published between 1957 and 2024. Reports were sourced from five world regions, with North America contributing the most (*n* = 26; 47.3%), followed by South America (*n* = 15; 27.3%), Africa (*n* = 7; 12,7%), Asia (*n* = 6; 10.9%), and Oceania (*n* = 1; 1.8%). The United States of America represented 43.6% of cases (*n* = 24), while other common contributors included Brazil 23.6% (*n* = 13), and South Africa 9.1% (*n* = 5), and other countries with fewer occurrences, such as Japan, Madagascar, Mexico, Australia, China, India, Malaysia, Peru, Thailand, and Venezuela. Regarding etiological agents, *Sporothrix schenckii* and/or *S. schenckii sensu stricto* was the most frequently reported species, particularly in earlier decades, whereas *S. brasiliensis* predominated in more recent reports, especially in South America.

### 3.3. Patient Demographics and Exposure

Patients aged 40–59 years old made up the largest percentage of affected patients (41.8%), followed by those aged 19–39 years old (34.5%). Individuals aged 60 years or older, as well as children and adolescents (0–18 years), accounted for 10.9% and 12.7% of the cases, respectively. By sex, men represented most cases (74.5%), compared to women (25.4%).

Regarding the route of transmission, saprophytic (69%) was predominant, while zoonotic transmission was reported in 16.3% of cases, mainly in Brazil, Venezuela, and Thailand. Some cases did not specify the transmission. The first zoonotic case was documented in 1992. Exposure through occupation or recreation was recorded in 78.1% of the total, frequently described in vague terms, with occasional occupations such as florist, gardener, farmer, construction worker, or landscaper.

### 3.4. Clinical Forms and Sporothrix Species Distribution

The primary causative agent of unexpected sporotrichosis was *Sporothrix schenckii*/*Sporothrix schenckii sensu stricto* (*n* = 48; 87.3%), while *S. brasiliensis* (*n* = 5; 9.1%) and *S. globosa* (*n* = 1; 1.8%) were noted exclusively in publications from after 2018. One recent case in the dataset was classified as *Sporothrix* spp. (untyped). Regarding the clinal forms, the most reported were osteoarticular (30.9%) and systemic (27.2%), followed by disseminated cutaneous (20%). Less common presentations included mucosal (14.51%) and pulmonary forms (7.2%).

**Figure 2:**
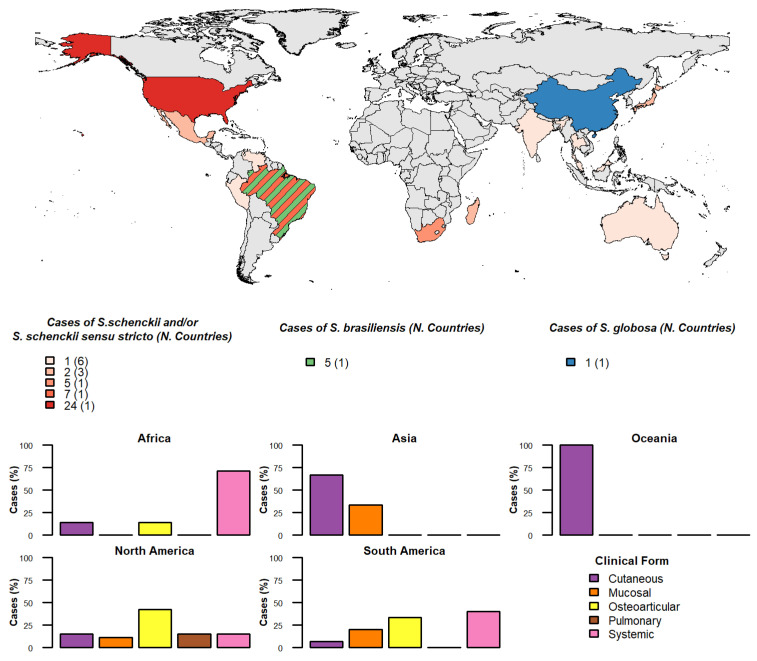
Geographical distribution of *Sporothrix* species and clinical forms among cases of “unusual sporotrichosis”. The world map illustrates the distribution of reported cases according to the identified *Sporothrix* species, with colours indicating *S. schenckii* and/or *Sporothrix schenckii sensu stricto* (red tones), *S. brasiliensis* (green), and *S. globosa* (blue). Countries with reports involving more than one *Sporothrix* species are shown with hatched patterns. Bar charts depict the percentage distribution of clinical forms (cutaneous, mucosal, osteoarticular, pulmonary, and systemic) stratified by region. The total number of cases analyzed per region was as follows: North America (*n* = 26), South America (*n* = 15), Africa (*n* = 7), Asia (*n* = 6), and Oceania (*n* = 1). Differences in the distribution of clinical forms across regions were assessed using Fisher’s Exact test (*p* = 0.0098).

As shown in Figure 2, the distribution of clinical forms varied significantly across regions (*p* = 0.0098). Across Africa, most cases were systemic (71.4%), with disseminated cutaneous and osteoarticular forms occurring less frequently (14.3% each). All isolates identified in this world region corresponded exclusively to *S. schenckii*/*S. schenckii sensu stricto* (100%).

In contrast, reports from Asia described only disseminated cutaneous (66.7%) and mucosal (33.3%) forms. No osteoarticular, pulmonary, or systemic cases were documented. Species identification confirmed *S. schenckii*/*S. schenckii sensu stricto* as the predominant agent (83.3%), while *S. globosa* accounted for 16.7%. Oceania contributed a single case, characterized by the disseminated cutaneous form, also attributed to *S. schenckii* (100%).

Moving to North America, osteoarticular was the most common form (42.3%), followed by pulmonary and systemic (15.4% each). Disseminated cutaneous and mucosal forms were less frequent, accounting for 11.5% and 15.4% respectively, of cases. All isolates from North America were identified as *S. schenckii*/*S. schenckii sensu stricto* (100%).

In South America, however, most cases were osteoarticular (33.3%) and systemic (40%), followed by mucosal (20%) and disseminated cutaneous (6.7%) forms. Pulmonary cases were not reported in this region. Regarding species distribution, *S. schenckii* was the most frequent (64.3%), while *S. brasiliensis* was also documented (35.7%), which was restricted to this world region.

Unusual clinical presentations of *Sporothrix* infections were equally distributed within the age groups (*p* = 0.1472). In children and adolescents (0–18 years), systemic and disseminated cutaneous forms were equally frequent, each representing 28.6% of cases; mucosal forms represented the major form with 42.9%. Among adults aged 19–39 years, systemic form was predominant (36.8%), followed by osteoarticular (31.6%), pulmonary form (15.8%), and disseminated cutaneous (10.5%). Patients aged 40–59 years were primarily affected by the osteoarticular form (39.1%), followed by systemic (26.1%) and disseminated cutaneous (17.4%) forms, with fewer cases of mucosal (13.0%) and pulmonary (4.3%) forms. In older adults (≥60 years), disseminated cutaneous forms were the most common (50%), followed by osteoarticular (33.3%) and mucosal (16.7%) forms, while no systemic or pulmonary cases were reported.

Among women, mucosal (35.7%) and disseminated cutaneous (21.4%) forms were followed by osteoarticular (21.4%) and systemic (21.4%) manifestations while the pulmonary form was not observed. Male patients, on the other hand, were mainly affected by osteoarticular (34.1%) and systemic (29.3%) forms, with disseminated cutaneous (19.5%), pulmonary (9.8%), and mucosal (7.3%) reported (*p* = 0.1414).

A significant temporal shift in clinical presentation was observed (*p* = 0.0074). In the first period (1956–1989), the osteoarticular form was the predominant form (46.2%), while the systemic (19.2%), disseminated cutaneous (15.4%), and pulmonary (15.4%) forms were less frequent, and the mucosal form was rare (3.8%). In contrast, during the second period (1990–2024), systemic forms became more prominent (34.5%), accompanied by increases in disseminated cutaneous and mucosal forms (24.1% each). Osteoarticular cases declined substantially (17.2%), and the pulmonary form was no longer reported.

### 3.5. Transmission Routes and Diagnostic Approaches

**Figure 3:**
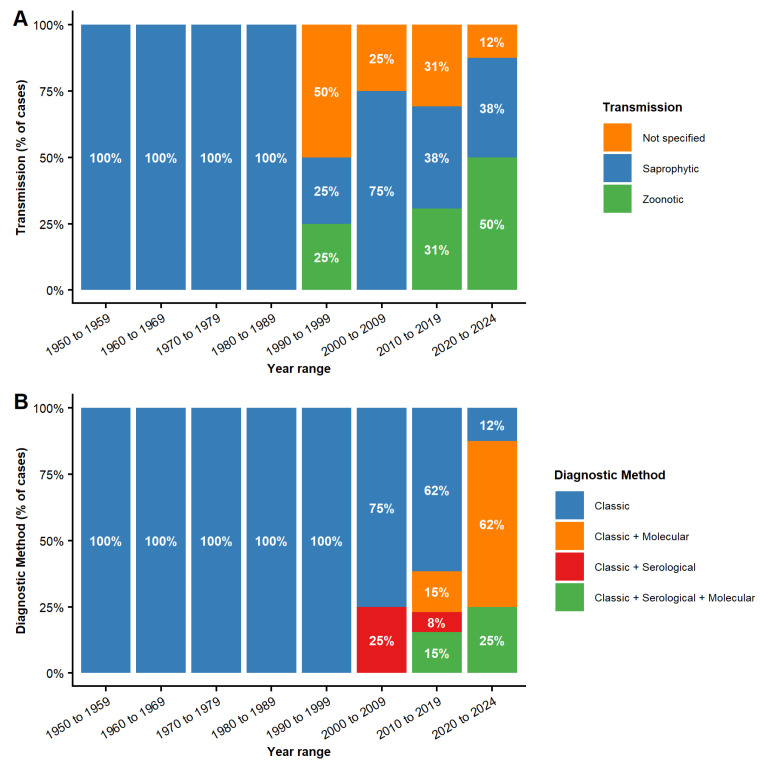
Distribution of transmission routes and diagnostic methods over time. (**A**) Temporal trends in transmission routes across predefined year ranges, showing the decline of exclusively saprophytic cases and the progressive emergence of zoonotic transmission. (**B**) Evolution of diagnostic approaches across the same periods, with classical methods predominating and a gradual incorporation of serological and molecular tools in combined diagnostic strategies. Bars represent the percentage of cases within each time period. The total number of cases analyzed in each period was of 26 patients from 1956 to 1989 and 29 patients from 1990 to 2024.

The analysis of transmission routes revealed a temporal shift (Figure 3A). In the earliest decades, cases were exclusively saprophytic. From the 1990s onwards, zoonotic transmission began to emerge and gradually increased, becoming more frequent than saprophytic in the most recent period.

Diagnostic methods followed a similar path of gradual diversification (Figure 3B). For decades, classical methods such as fungal culture, microscopy, and histopathology were used almost exclusively. Culture remained the backbone of diagnosis, whether applied alone or together with other classical techniques. From the 2000s onwards, however, new strategies began to emerge, integrating serological and classical methods, although classical methods alone still predominated.

The 2010s marked a notable diversification, with the prevalence of classical methods decreasing. During this decade, the use of “Classic + Molecular” and “Classic + Serological + Molecular” methods was adopted, while the “Classic + Serological” combination was also used. This trend of consolidating combined methods continued in the most recent period (2020–2024), indicating a decisive shift toward more robust diagnoses.

### 3.6. Therapeutic Strategies

A wide variety of therapeutic strategies were reported, including antifungals, antibacterials, surgical interventions, and adjuvant therapies. Polyenes were the most frequently used agents, mainly amphotericin B, which was employed especially in systemic and osteoarticular cases. Potassium iodide was also widely employed, particularly in cutaneous and systemic forms. Azoles represented by miconazole, itraconazole, and voriconazole were most often used in systemic and disseminated cutaneous cases.

Antibacterials were used in all clinical forms. This group included broad-spectrum antibiotics such as aureomycin, streptomycin, terramycin, penicillin (including benzathine), oxytetracycline, polymyxin B, neomycin sulfate, and levofloxacin, as well as isoniazid and rifampicin. Other antimicrobials, including hydroxystilbamidine, mapharsen, and flucytosine, appeared less frequently. Importantly, information on confirmed bacterial coinfections was rarely reported in the included studies. In most cases, antibacterial therapy appeared to have been prescribed empirically prior to mycological confirmation of sporotrichosis, reflecting initial misdiagnosis rather than documented bacterial coinfection.

Surgical procedures were most often associated with osteoarticular form, followed by pulmonary, mucosal, and disseminated cutaneous forms; no systemic cases were treated surgically. Reported procedures included resection, synovectomy, medial meniscectomy, arthrodesis, orchiectomy, dacryocystorhinostomy and bronchoscopy. Adjuvant and supportive therapies included immunotherapy with autogenous vaccine, albuterol, prednisolone, dexamethasone, and nonsteroidal anti-inflammatory drugs, often combined with antifungal regimens. Most reports involved mucosal and osteoarticular forms, with a smaller number in systemic cases. No surgical intervention was recorded for disseminated cutaneous or pulmonary forms.

Combined therapeutic strategies involving different drug categories were also identified. The most frequent associations were polyenes with azoles, surgery with azoles, and surgery with polyenes. Other combinations included potassium iodide with polyenes, azoles, or other antifungals, as well as regimens in which antibacterials were administered together with potassium iodide, polyenes, azoles, or surgical procedures. In some instances, more complex schemes were reported, integrating four or more categories such as antibacterials, potassium iodide, polyenes, other antifungals, and adjuvant therapies. These findings demonstrate that, in addition to the use of individual agents, therapeutic combinations also formed part of the treatment spectrum.

### 3.7. Outcomes

**Figure 4:**
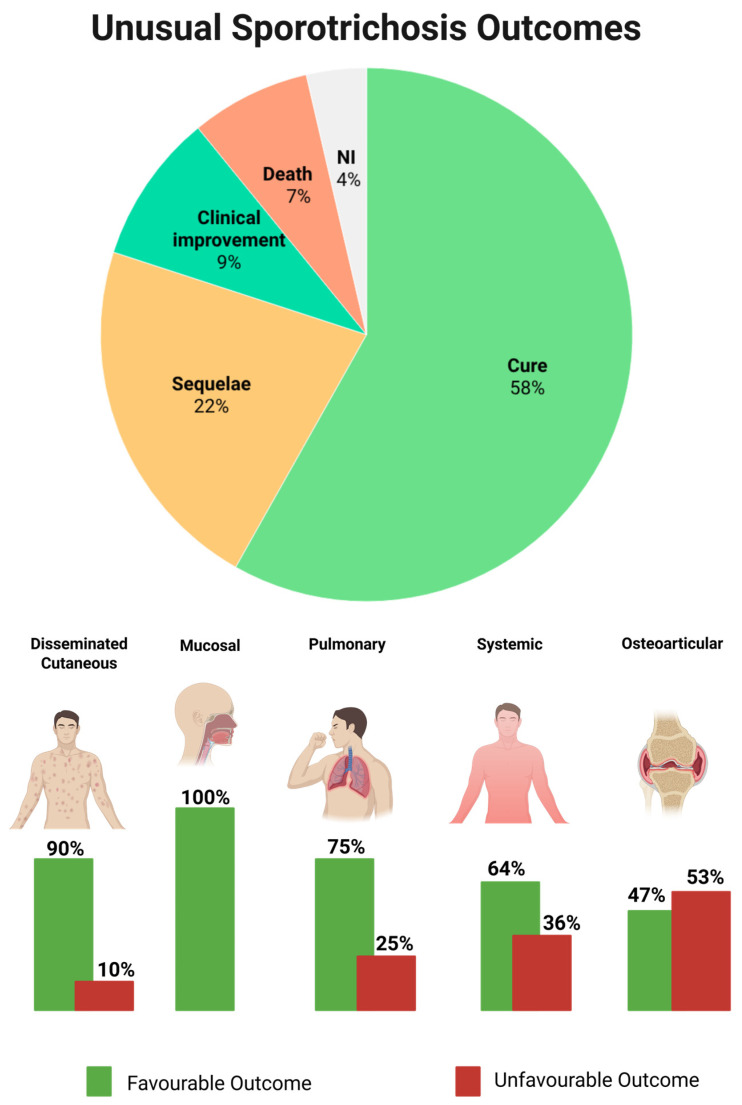
Clinical outcomes of unusual sporotrichosis and their distribution across clinical forms. The upper panel shows the overall distribution of clinical outcomes, including cure, sequelae, clinical improvement, death, and cases with no information (NI). The lower panel illustrates outcomes stratified by clinical form, comparing favourable outcomes (green bars) and unfavourable outcomes (red bars) across different clinical presentations.

The most common outcome was cure, observed in 58.1% of cases, while sequelae were reported in 21.8%. Clinical improvement without complete resolution accounted for 9%, and death occurred in 7.2% of patients (Figure 4).

When stratified by clinical form, patterns emerged (*p* = 0.0435). The disseminated cutaneous form showed the most favourable prognosis, with cure or clinical improvement in 90% of cases, and sequelae in only one case (10%). Mucosal form was also associated with excellent outcomes, as all reported patients achieved full recovery. Pulmonary forms had a predominance of cure or improvement (75%), although isolated fatal cases were documented. In systemic form, outcomes were less favourable, with cure or improvement in 64.3% of cases and 35.7% progressing to death or sequelae. The most severe prognosis was linked to osteoarticular form (Figure 4), where more than half of the patients (52.9%) developed death or sequelae, highlighting the chronic and disabling nature of this form.

Overall, “unusual sporotrichosis” predominantly affected immunocompetent adults, frequently involved extracutaneous sites, and was associated with delayed diagnosis, prolonged treatment, and unfavourable outcomes, particularly in disseminated forms.

## 4. Discussion

This systematic review synthesized 55 cases of “unusual sporotrichosis” published between 1956 and 2024, selected through rigorous eligibility criteria designed to capture uncommon presentations of the mycosis, revealing that clinically severe and atypical manifestations of sporotrichosis are consistently reported even in the absence of classical risk factors. By deliberately focusing on this subset, the review highlights a dimension of sporotrichosis that is likely underrecognized in routine clinical practice. In this study, we propose the definition of “unusual sporotrichosis” as clinical forms that diverge from the classical lymphocutaneous or fixed-cutaneous patterns and occur in individuals without any type of immunosuppression/diabetes, or alcohol abuse. Accordingly, cases involving the typical lymphocutaneous and fixed-cutaneous forms, which are the most frequent both in high-risk hosts and in the general population affected by *Sporothrix* [60,61], were not included. Likewise, cases in which clinical presentation was influenced by infectious or drug-induced immunosuppression, chronic alcohol abuse, or other comorbidities that could distort interpretation were excluded. Rather than representing the full clinical spectrum of sporotrichosis, this dataset intentionally isolates a restricted yet clinically meaningful subset of cases, allowing focused examination of unusual anatomical tropism of its etiological agents, nontraditional routes of infection, and disease severity occurring in immunocompetent hosts. These characteristics highlight the clinical complexity of this subset and support the need for heightened diagnostic awareness beyond the classical cutaneous paradigms.

Importantly, the designation “unusual” should be interpreted within a dual conceptual framework. On one hand, it reflects genuinely atypical clinical phenotypes, involving rare anatomical sites, nontraditional routes of infection, or increased morbidity when compared with classical cutaneous forms. On the other, it inevitably incorporates elements of publication bias, as severe, refractory, or anatomically uncommon cases are more likely to be reported in the literature. These two dimensions are not mutually exclusive and likely coexist within the cases analyzed in this review. In this context, careful appraisal of the methodological quality and reporting standards of the included studies is essential for a balanced interpretation of the evidence.

The critical appraisal of the included articles revealed heterogeneity in methodological approaches, especially among older publications, which frequently lacked detailed information on clinical context and patient inclusion. Even so, all articles, including those of lower methodological robustness, were retained because they offer valuable epidemiological and clinical insights into the historical evolution of unexpected human sporotrichosis, as noted in other systematic reviews [4,62]. In contrast, more recent publications reflect substantial improvements in quality, with more precise descriptions of demographic data, clinical course, and diagnostic methods, aligned with advances in reporting guidelines and the adoption of refined molecular techniques. Including all eligible studies expanded our ability to interpret the findings and highlights recent improvements in standardization and diagnostic accuracy that are changing the way that the disease is understood.

“Unusual sporotrichosis”, as defined in this review under strict inclusion criteria, demonstrated worldwide distribution across five world regions. At the country level, the United States of America (USA) and Brazil accounted for the highest number of cases. The predominance of USA reports should not be interpreted solely as a reflection of disease incidence; rather, it likely reflects the broader availability of diagnostic resources, a longer-standing tradition of scientific reporting, and structured epidemiological surveillance, all factors that facilitate publication of cases in internationally indexed journals [13,14].

Brazil plays a central epidemiological role owing to the prominence of zoonotic transmission in human sporotrichosis, strongly influenced by the outbreak that began in the 1990s in Rio de Janeiro. The disease is now considered a neglected zoonosis and constitutes a national hyperendemic condition, raising concern due to the emergence of *Sporothrix brasiliensis* as a highly virulent zoonotic agent expanding across Latin America. Thus, the substantial number of unexpected cases identified in Brazil in this review is unsurprising. The incorporation of molecular techniques in recent studies enabled reclassification of isolates previously assigned generically to *S. schenckii*, revealing the predominance of *S. brasiliensis* in human cases even before the current epizootic and hyperendemic period, thereby expanding understanding of its distribution [8,62]. This methodological advance helps explain the increased Brazilian representation in recent series and illustrates how diagnostic progress has reshaped the epidemiological landscape.

South Africa, the third most represented country, has long been a focal point for clinical mycology, though data quality remains contingent on expanded confirmatory methods and improved surveillance [63]. Meanwhile, isolated cases reported in Japan, Madagascar, Mexico, Australia, China, India, Malaysia, Peru, Thailand, and Venezuela demonstrate that “*unusual sporotrichosis*” indeed has an intercontinental distribution. However, the limited number of publications from these regions likely reflects underdiagnosis, laboratory limitations, and publication bias rather than the true absence of cases, a recurring pattern among neglected fungal diseases [15,62]. Taken together, these findings indicate that the world region variation in clinical presentations observed in this review reflects a composite effect of regional epidemiological characteristics, differential access to diagnostic resources, and publication-related case-mix, rather than isolated biological differences among populations.

Although *S. brasiliensis* is recognized as the most virulent species within the *Sporothrix* complex [64], *S. schenckii*/*S. schenckii sensu stricto* was the most frequently reported in our dataset. This finding should be interpreted with caution, as a substantial proportion of the included studies were published prior to the taxonomic recognition of cryptic species within the *S. schenckii* complex and relied exclusively on classical phenotypic identification methods. Therefore, cases reported as *S. schenckii* in older publications may represent members of the *S. schenckii* species complex rather than *S. schenckii sensu stricto*. The role of *S. brasiliensis* in “*unusual sporotrichosis*” is likely underestimated, partly because species-level delineation within what was previously considered a single genus began only in 2007–2010 [65], and was consolidated in 2016 [2]; and partly because molecular diagnostic techniques, essential for accurate species identification, are not routinely used.

Indeed, recent studies employing sequencing and confirmatory PCR targeting calmodulin and β-tubulin genes demonstrated that an important number of older isolates previously classified as *S. schenckii* were in fact *S. brasiliensis* [66,67]. Thus, part of the epidemiological gap identified in this review likely reflects underdiagnosis rather than the true absence of the species, further emphasizing the need to expand molecular tools in mycological surveillance to better understand zoonotic sporotrichosis and the relevance of *S. brasiliensis* in unusual human disease. Accurate identification at the species level extends beyond taxonomic refinement and has meaningful clinical and public health implications. Different *Sporothrix* species have been associated with distinct virulence profiles, transmission dynamics, and clinical trajectories, which may influence disease severity, therapeutic response, and the risk of complications. Infections caused by *S. brasiliensis* have been consistently linked to higher fungal burden, enhanced tissue invasiveness, and a greater propensity for severe extracutaneous involvement [68], underscoring the relevance of early species identification for risk stratification, prognostic assessment, and surveillance planning. Importantly, the absence of species-level identification in many reports among the selected in this review should not be interpreted as evidence of species absence, but rather as a reflection of historical and regional limitations in diagnostic capacity, especially in older studies relying exclusively on classical methods.

The marked predominance of male patients and the average age (43.02 years) reflect recurrent patterns in sporotrichosis studies, typically linked to occupational and recreational exposures to soil and vegetation. This demographic profile aligns with reports from diverse endemic areas, where gardening, agriculture, and construction are major risk factors [8,24]. However, it contrasts with the Brazilian hyperendemic scenario driven by *S. brasiliensis,* in which women are more frequently affected due to their predominant role in caring for infected cats [69,70,71].

Regarding transmission, the predominance of sapronotic inoculation in this review reinforces the classical interpretation that environmental inoculation is the major route of infection. Nevertheless, zoonotic outbreaks in South America, particularly involving *S. brasiliensis* in Brazil, are reshaping this understanding, with thousands of human cases linked to contact with infected felines [13,72]. Reports from Thailand [73] that zoonotic transmission also occurs outside Brazil support the notion that this pathway is increasing globally. In addition, many reports provide only vague occupational descriptions (“gardener,” “florist,” “rural worker”), and some do not specify any probable source of infection. These limitations hinder precise analysis of exposure–risk associations and reflect broader methodological deficiencies in the literature. Although sporotrichosis became the first nationally notifiable fungal disease in Brazil in 2025 [74] structured surveillance remains limited, contributing to the underestimation of both sapronotic and zoonotic cases [4]. These findings point to the relevance of exposure-related determinants in shaping the clinical presentation of unusual sporotrichosis, while also highlighting important gaps in case reporting. In many studies, key epidemiological variables, such as the nature of animal contact, history of scratches or bites, occupational exposure, environmental activities, and the interval between symptom onset and diagnosis, were incompletely described or entirely absent. The systematic reporting of these elements would not only improve epidemiological interpretation but also facilitate earlier clinical suspicion, more accurate risk assessment, and the development of targeted prevention and surveillance strategies, particularly in settings where zoonotic transmission is emerging or underrecognized. Among all the 55 cases described in the present systematic review, osteoarticular and systemic presentations were the most common “*unusual sporotrichosis*”, followed by disseminated cutaneous, mucosal, and, less frequently, pulmonary forms. This pattern illustrates the clinical diversity of unexpected sporotrichosis and reinforces that infection extends beyond subcutaneous tissue, supporting the proposition that it should be categorized as a “traumatic implantation mycosis” rather than strictly “subcutaneous.” The exclusion of immunosuppressed patients underscores the significance of severe disease manifestation in otherwise healthy individuals, thereby highlighting the considerable pathogenic potential of *Sporothrix* spp. In this context, an additional line of investigation involves the possibility that polymorphisms in human immune response genes (e.g., Toll-like receptors, cytokines) confer differential genetic susceptibility, as shown for other infectious diseases [75,76].

Regional variations pointed out the influence of local epidemiology and species diversity. In Africa and North America, systemic and osteoarticular forms caused by *S. schenckii*/*S. schenckii sensu stricto* predominated, consistent with classical sapronotic profiles. In Asia, disseminated cutaneous forms associated with *S. globosa*, a less virulent species, were more frequent [77], particularly in northeastern China, where farming-related exposure to corn straw underlies a sapronotic hyperendemic pattern [78]. In South America, the coexistence of *S. schenckii* and *S. brasiliensis* corresponds to a broader spectrum of unexpected manifestations, including frequent osteoarticular involvement and aggressive systemic forms [5,20], supporting the need for active surveillance for articular and disseminated presentations in the region.

Historical comparison revealed a marked shift in the clinical spectrum. Until the 1980s, osteoarticular forms predominated, reflecting classical sapronotic transmission and the near-exclusive identification of *S. schenckii*. From the 1990s onward, systemic and mucosal forms became more common, concurrent with the emergence of *S. brasiliensis* in South America, a more virulent species strongly associated with zoonotic transmission and severe disease [72]. This change likely reflects, at least in part, genuine epidemiological transformations, including altered exposure patterns and the expansion of zoonotic transmission. This temporal shift is also attributable to the adoption of molecular diagnostics, which enabled reclassification of older isolates and recognition of species diversity within the *Sporothrix* complex [62,67]. Expanded surveillance and scientific interest in the zoonosis further increased detection and reporting of unexpected cases that were likely overlooked in earlier decades [79], contributing to an apparent amplification of severe and atypical presentations in contemporary literature.

The evolution of diagnostic approaches parallels these changes. For much of the 20th century, reliance on mycological culture, microscopy, and histopathology limited species-level precision and likely contributed to overrepresentation of *S. schenckii* and underrecognition of unexpected forms or emerging species like *S. brasiliensis* [80]. From the early 2000s onward, serological testing became more widely used, and its integration with molecular tools strengthened diagnostic accuracy and enabled retrospective reclassification of isolates, revealing previously unrecognized diversity [62,79]. Uneven regional adoption of these methods, greater in South America and Asia, and more limited in North America and Africa, likely affects the spectrum of reported cases, reinforcing that temporal trends in clinical presentation reflect both real changes in disease dynamics and shifts in diagnostic and publication practices.

Therapeutic approaches varied widely, including antifungals, antibiotics, surgical procedures, and adjunctive therapies. Amphotericin B was predominantly used for systemic and osteoarticular forms, while itraconazole was preferred for disseminated cutaneous disease, consistent with current recommendations [8,81]. Potassium iodide appeared in some cases, mainly cutaneous–disseminated or systemic, reflecting its continued use in resource-limited settings due to its low cost and operational feasibility. Beyond therapeutic heterogeneity, a critical and recurrent pattern identified in this review was the frequent use of antibacterials across clinical forms, indicating persistent diagnostic delays or misdiagnosis, leading to initial treatment as a bacterial infection. Rather than representing isolated prescribing errors, this pattern likely reflects the clinical mimicry of sporotrichosis, which may resemble bacterial osteomyelitis, septic arthritis, chronic sinusitis, ocular infections, or systemic inflammatory conditions. In Rio de Janeiro, nearly 90% of patients referred to the reference centre arrived on topical or oral antibacterial therapy [82]. The widespread use of antibacterials before mycological investigation emerges as a practical marker of delayed diagnosis which can prolong the course of the disease and contribute to progression to severe forms and the need for surgical intervention, particularly in osteoarticular cases [20,22,24]. These findings suggest that sporotrichosis should be actively considered in chronic osteoarticular infections unresponsive to antibacterials, unexplained mucosal involvement of the nasal or ocular regions, and systemic inflammatory presentations without a clear bacterial focus.

The acknowledged heterogeneity of therapeutic regimens for sporotrichosis, disease severity, and follow-up duration prevents any inference regarding comparative treatment efficacy or prognostic superiority among antifungal strategies [83]. Such limitations extend beyond unusual sporotrichosis cases, mirroring wider challenges in defining evidence-based guidelines for rare and atypical fungal infections. Directives for these mycoses often suffer from publication biases in case reports and limited series, toughening reliable assessments of incidence, clinical diversity, and prognosis. Comparable variability arises in atypical forms of prevalent mycoses like candidiasis, aspergillosis, and cryptococcosis, where trials and foundational studies prioritize acknowledged scenarios (e.g., Candida-driven candidemia, *Aspergillus*-associated pulmonary disease, and Cryptococcus-induced meningitis) [84]. In this context, the heterogeneity of diagnostic approaches, therapeutic regimens, and reported outcomes observed in “unusual sporotrichosis” should be understood as a reflection of a not-yet-recognized clinical entity.

Most “unusual sporotrichosis” patients, as defined in this systematic review, ultimately achieved cure or clinical improvement, especially those with disseminated cutaneous or mucosal forms, which had good prognoses. However, outcomes were less favourable for systemic and particularly for the osteoarticular forms, in which over half of patients developed disabling sequelae or died. These findings reinforce the persistent therapeutic challenges of bone sporotrichosis, especially in infections caused by *S. brasiliensis* [19,82,85,86,87].

The occurrence of severe forms of sporotrichosis in immunocompetent individuals raises important questions regarding the mechanisms underlying disease progression in this subset of patients. Rather than a single factor, our findings support a multifactorial model, encompassing several non-mutually exclusive hypotheses. First, the route and depth of inoculation, along with fungal burden, likely drive severity: traumatic implantation into deeper tissues (e.g., periarticular regions) facilitates local persistence, dissemination, chronic inflammation, and tissue destruction—consistent with the predominance of osteoarticular and systemic forms, frequent surgical interventions, and high rates of sequelae in this review. Second, pathogen-related factors, such as species-specific virulence, contribute markedly; infections by *Sporothrix brasiliensis* were disproportionately linked to extracutaneous manifestations, aligning with evidence of its enhanced invasiveness [66]. Third, host factors and diagnostic delays exacerbate progression: subtle variations in immune responses, such as those encoded by polymorphic genes, combined with hard-to-detect anatomical sites, allow fungal persistence, while initial antibacterial therapies—common before mycological confirmation—promote extension, irreversible damage, and poorer outcomes. This framework explains the emergence of disabling or fatal ‘unusual sporotrichosis’ in immunocompetent hosts, as documented here, and underscores the need for targeted research into these interactions.

This review has limitations that arise both from the nature of the available evidence and from the methodological constraints inherent to the review process itself. With respect to the body of evidence, the findings are predominantly derived from case reports and small case series, which are inherently subject to publication bias, incomplete clinical and epidemiological reporting, heterogeneous follow-up, and limited external validity. Historical changes in taxonomy and diagnostic practices, including the absence of species-level identification in many earlier studies, further constrain interpretation, while the operational definition of “unusual sporotrichosis” necessarily captures an extreme subset of the disease spectrum. Together, these factors preclude quantitative synthesis and comparative inferences regarding treatment efficacy or prognosis. Regarding the review process, although a comprehensive and systematic search strategy was applied, limitations related to database coverage, language restrictions, access to full-text articles, and indexation practices may have resulted in the omission of relevant reports, particularly from regions with lower publication visibility. In addition, the heterogeneity of study designs, reporting standards, and outcome measures contributed to preclude meta-analysis, and required narrative synthesis and consensus-based decisions at multiple stages of study selection and data interpretation. Nevertheless, despite these limitations, the systematic and concept-driven approach adopted in this review allowed the consistent identification of recurring clinical patterns, exposure profiles, and disease trajectories that would be difficult to apprehend through isolated case reports alone.

Overall, this systematic review adopts the term “unusual sporotrichosis” as a descriptive and operational basis to support recognition of a specific subset of severe and/or atypical presentations reported in the literature. However, it is not intended to establish a new clinical classification but rather to complement existing frameworks—such as those proposed by Orofino-Costa et al. [81] 2017 and Ramírez-Soto [12] 2025—by highlighting cases that may be underrecognized in clinical practice. Hence, “unusual sporotrichosis” is a proposed model integrating severity, patterns, and host factors to organize a set of distinct cases without creating a new category. The possibility of designating “unusual sporotrichosis” as a clinical category will not stem solely from the proposition advanced in this article. Instead, the framework proposed herein requires rigorous validation through future studies that operationalize and test the definition delineated in our investigation.

The authors were motivated by the need to address emerging clinical scenarios that arose following the zoonotic sporotrichosis epidemic in Rio de Janeiro, Brazil. Furthermore, given its clinical classification, as outlined in this review, together with molecular species identification and the recent implementation of mandatory notification in Brazil, cases of “unusual sporotrichosis” caused by *S. brasiliensis* are likely to increase in forthcoming epidemiological studies.

## 5. Conclusions

Our research shows that “unusual sporotrichosis”—a term introduced in this review to describe clinical cases that differ from the typical lymphocutaneous or fixed-cutaneous patterns and occur in people without immunosuppression/comorbidities—can impact otherwise healthy individuals. It may present with severe, widespread, or atypical symptoms, which require clinicians to maintain a heightened awareness beyond standard risk profiles. Clinical evidence also indicates that osteoarticular sporotrichosis contributes to worse outcomes among immunocompetent individuals, including long-term sequelae and mortality. Integrated use of classical and molecular methods has improved diagnostic accuracy, clarified species-specific roles, and uncovered previously underestimated transmission routes. Nevertheless, important limitations persist, including underreporting of exposures, methodological heterogeneity, and diagnostic delays that lead to inappropriate therapeutic strategies. Clinically, early species-level identification through multimodal diagnostics and prompt initiation of targeted antifungal therapy, avoiding empirical antibiotic “therapeutic trials” when fungal infection is plausible, are essential. From a public health perspective, active surveillance, rigorous standardization of reporting, and expanded access to specialized diagnostics are crucial to reducing underdiagnosis, guiding prevention, and improving outcomes.

## Figures and Tables

**Table 1:** Demographic and study design information.

Citation	World Region/Country	Study Design	Cases in the Article	Unusual Cases	Age (Year) And Sex	Laboral/Recreation Exposure
Webster & Willander [50], 1957	North America/USA	Case report	1	1	30 (M)	YES
Garrett & Robbins [51], 1960	North America/USA	Series of Cases	8	2	13 (F) 10(F)	YES (2)
Lurie [53], 1963	Africa/South Africa	Series of Cases	5	4	43 (M)/50 (M)/45 (M)/38 (M)	YES (4)
Baum et al. [52], 1969	North America/USA	Series of Cases	4	1	34 (M)	YES
Urabe & Nagashima [49], 1970	Asia/Japan	Case report	1	1	65 (M)	YES
Winter & Pearson [48], 1972	North America/USA	Series of Cases	5	4	56 (M), 47 (F), 51 (M), 64 (M)	YES (3) + NI (1)
Serstock & Zinneman [47], 1975	North America/USA	Series of Cases	2	1	36 (M)	YES
Kluge &Hornick [46], 1976	North America/USA	Series of Cases	2	1	47 (M)	NI
Michelson [45], 1977	North America/USA	Case report	1	1	38 (M)	NI
Crout et al. [43], 1977	North America/USA	Series of Cases	7	3	51 (M)/54 (M)/25 (M)	YES (2) NI (1)
Beardmore [44], 1979	Oceania/Australia	Case report	1	1	41 (M)	YES
Matthews et al. [42], 1982	Africa/South Africa	Case report	1	1	56 (F)	YES
Shelley & Sica [41], 1983	North America/USA	Case report	1	1	39 (M)	YES
Friedman & Doyle [40], 1983	North America/USA	Series of Cases	11	4	71 (M)/54 (M)/25 (M)/ 52 (M)	YES (2) NI (2)
Pérez et al. [56], 1992	South America/ Venezuela	Case report	1	1	58 (M)	YES
Neafie & Marty [38], 1993	North America/USA	Case report	1	1	74 (M)	NI
Saaibi et al. [37], 1996	North America/USA	Case report	1	1	36 (F)	NI
Clay & Anand [36],1996	North America/USA	Case report	1	1	3 months (M)	NI
Khabie et al. [35], 2003	North America/USA	Case report	1	1	19 months (F)	YES
Curi et al. [34], 2003	South America/Brazil	Case report	1	1	18 (M)	NI
Appenzeller et al. [33], 2006	South America/Brazil	Series of Cases	2	2	47 (M)/35 (M)	YES (2)
Yap [32], 2011	Asia/Malaysia	Case report	1	1	70 (F)	YES
Romero-Cabello et al. [30], 2011	North America/Mexico	Case report	1	1	36 (M)	YES
Marques De Macedo et al. [54], 2015	South America/Brazil	Case report	1	1	9 (F)	YES
Ribeiro et al. [31], 2015	South America/Brazil	Case report	1	1	5 (M)	YES
Sanke et al. [29], 2016	Asia/India	Case report	1	1	54 (M)	YES
Ferreira et al. [28], 2016	South America/Brazil	Case report	1	1	35 (M)	YES
Hessler et al. [17], 2016	North America/USA	Case report	1	1	34 (M)	YES
Mialski et al. [26], 2018	South America/Brazil	Series of Cases	2	2	46 (M)/40 (M)	YES (2)
Rueda et al. [27], 2018	South America /Peru	Case report	1	1	42 (M)	YES
Sun et al. [26], 2018	Asia/Japan	Case report	1	1	34 (F)	NO
Furtado et al. [26], 2019	South America /Brazil	Series of Cases	2	2	50 (M), 46 (F)	YES (2)
Reinprayoon et al. [23], 2020	Asia/Thailand	Case report	1	1	42 (F)	YES
Sendrasoa et al. [22], 2021	Africa/Madagascar	Case report	1	1	34 (M)	YES
Martínez-Herrera et al. [21], 2021	North America/Mexico	Series of Cases	2	1	21 (M)	YES
Zhuang et al. [20], 2022	Asia/China	Case report	1	1	47 (M)	YES
Siqueira et al. [19], 2023	South America/Brazil	Series of Cases	2	2	78 (F), 37 (F)	YES (2)
Rakotoarisaona et al. [18], 2024	Africa/Madagascar	Case report	1	1	35 (M)	YES
Texeira et al. [55], 2024	South America/Brazil	Case report	1	1	25 (F)	YES

**Notes:** USA = United States of America; M = male; F = female. “Case report” = single case report; “Series of cases” = case series. “Unusual cases” refers to the number of cases described as atypical by the authors. “NI” indicates data not informed in the original article. Numbers in parentheses represent the number of cases in the article with that specific information (e.g., YES (2) = two cases with reported exposure).

## Data Availability

The data that support the findings of this study are available in the Open Science Frame (www.osf.io, accessed on 21 December 2025) DOI: 10.17605/OSF.IO/Q6UWB registration protocol and from the corresponding authors upon reasonable request.

## Supplementary Materials

The following supporting information can be downloaded at: https://www.mdpi.com/article/10.3390/jof12020155/s1, File S1: PRISMA 2020 Checklist.

## Abbreviations

The following abbreviations are used in this manuscript:

AIDS	Acquired Immunodeficiency Syndrome
BVS	Virtual Health Library (Biblioteca Virtual em Saúde)
HIV	Human Immunodeficiency Virus
JBI	Joanna Briggs Institute
LILACS	Latin American and Caribbean Health Sciences Literature
MeSH	Medical Subject Headings
OSF	Open Science Framework
PICO	Population, Intervention, Comparison, Outcome
PRISMA	Preferred Reporting Items for Systematic Reviews and Meta-Analyses
RJ	Rio de Janeiro
spp.	Species (plural)
USA	United States of America

## Appendix A

**Table A1:** Risk of Bias.

**Single Case Reports**		
**Authors**	**%**	**Risk of Bias/Methodological Quality**
**Webster & Willander [50], 1957**	100	Low risk of bias/High methodological quality
**Urabe & Nagashima [49], 1970**	87.5	Low risk of bias/High methodological quality
**Michelson [45], 1977**	93.7	Low risk of bias/High methodological quality
**Beardmore [44], 1979**	100	Low risk of bias/High methodological quality
**Matthews et al. [42], 1982**	100	Low risk of bias/High methodological quality
**Shelley & Sica [41], 1983**	100	Low risk of bias/High methodological quality
**Pérez et al. [56], 1992**	87.5	Low risk of bias/High methodological quality
**Neafie & Marty [38], 1993**	93.7	Low risk of bias/High methodological quality
**Saaibi et al. [37], 1996**	100	Low risk of bias/High methodological quality
**Clay & Anand [36],1996**	93.7	Low risk of bias/High methodological quality
**Khabie et al. [35], 2003**	100	Low risk of bias/High methodological quality
**Curi et al. [34], 2003**	92.8	Low risk of bias/High methodological quality
**Yap [32], 2011**	93.7	Low risk of bias/High methodological quality
**Romero-Cabello et al. [30], 2011**	93.8	Low risk of bias/High methodological quality
**Marques De Macedo et al. [54], 2015**	81.2	Low risk of bias/High methodological quality
**Ribeiro et al. [31], 2015**	81.2	Low risk of bias/High methodological quality
**Sanke et al. [29], 2016**	81.2	Low risk of bias/High methodological quality
**Ferreira et al. [28], 2016**	87.6	Low risk of bias/High methodological quality
**Hessler et al. [17], 2016**	87.5	Low risk of bias/High methodological quality
**Rueda et al. [27], 2018**	93.8	Low risk of bias/High methodological quality
**Sun et al. [26], 2018**	87.6	Low risk of bias/High methodological quality
**Reinprayoon et al. [23], 2020**	100	Low risk of bias/High methodological quality
**Sendrasoa et al. [22], 2021**	93.8	Low risk of bias/High methodological quality
**Zhuang et al. [20], 2022**	100	Low risk of bias/High methodological quality
**Rakotoarisaona et al. [18], 2024**	93.7	Low risk of bias/High methodological quality
**Case Series**		
**Authors**	**%**	**Risk of Bias/Methodological Quality**
**Garrett & Robbins [51], 1960**	50	Moderate risk of bias
**Lurie [53], 1963**	72.2	Low risk of bias/High methodological quality
**Baum et al. [52], 1969**	72.2	Low risk of bias/High methodological quality
**Winter & Pearson [48], 1972**	66.6	Moderate risk of bias
**Serstock & Zinneman [47], 1975**	77.7	Low risk of bias/High methodological quality
**Kluge & Hornick [46], 1976**	77.7	Low risk of bias/High methodological quality
**Crout et al. [43], 1977**	72.2	Low risk of bias/High methodological quality
**Friedman & Doyle [40], 1983**	77.7	Low risk of bias/High methodological quality
**Appenzeller et al. [33], 2006**	77.7	Low risk of bias/High methodological quality
**Mialski et al. [26], 2018**	88.8	Low risk of bias/High methodological quality
**Furtado et al. [26], 2019**	83.3	Low risk of bias/High methodological quality
**Martínez-Herrera et al. [21], 2021**	82.6	Low risk of bias/High methodological quality
**Siqueira et al. [19], 2023**	72.2	Low risk of bias/High methodological quality

## Appendix B

**Table A2:** Individual patient clinical information.

Citation	World Region/Country	Age	Sex	Exposure	Diagnostic	Species	Transmission	Clinical Form	Treatment	Outcome
Webster & Willander [50], 1957	North America/USA	30	M	Yes	Classic	*S. schenckii*	Saprophytic	Osteoarticular	Potassium Iodide + Other Antifungals + Surgical	Cure
Garrett & Robbins [51], 1960	North America/USA	13	F	Yes	Classic	*S. schenckii*	Saprophytic	Disseminated Cutaneous	Potassium Iodide	Cure
Garrett & Robbins [51], 1960	North America/USA	10	F	Yes	Classic	*S. schenckii*	Saprophytic	Disseminated Cutaneous	Potassium Iodide	Cure
Lurie [53], 1963	Africa/South Africa	43	M	Yes	Classic	*S. schenckii*	Saprophytic	Systemic	Potassium Iodide	Cure
Lurie [53], 1963	Africa/South Africa	50	M	Yes	Classic	*S. schenckii*	Saprophytic	Systemic	Potassium Iodide	Cure
Lurie [53], 1963	Africa/South Africa	45	M	Yes	Classic	*S. schenckii*	Saprophytic	Osteoarticular	Potassium Iodide + Other Antifungals	Cure
Lurie [53], 1963	Africa/South Africa	38	M	Yes	Classic	*S. schenckii*	Saprophytic	Systemic	Antibacterials + Potassium Iodide + Other Antifungals + Adjuvants/Others	Cure
Baum et al. [52], 1969)	North America/USA	34	M	Yes	Classic	*S. schenckii*	Saprophytic	Pulmonary	Antibacterials + Polyenes + Surgical +Potassium Iodide.	Cure
Urabe & Nagashima [49], 1970	Asia/Japan	65	M	Yes	Classic	*S. schenckii*	Saprophytic	Disseminated Cutaneous	Antibacterials + Other Antifungals + Potassium Iodide	Sequelae
Winter & Pearson [48], 1972	North America/USA	56	M	Yes	Classic	*S. schenckii*	Saprophytic	Osteoarticular	Potassium Iodide	Cure
Winter & Pearson [48], 1972	North America/USA	47	F	Yes	Classic	*S. schenckii*	Saprophytic	Osteoarticular	Polyenes	Clinical improvent
Winter & Pearson [48], 1972	North America/USA	51	M	Yes	Classic	*S. schenckii*	Saprophytic	Osteoarticular	Polyenes	Clinical improvent
Winter & Pearson [48], 1972	North America/USA	64	M	NI	Classic	*S. schenckii*	Saprophytic	Osteoarticular	Polyenes	Disease persists
Serstock & Zinneman [47], 1975	North America/USA	36	M	Yes	Classic	*S. schenckii*	Saprophytic	Pulmonary	Polyenes + Surgical+ Antibacterials	Cure
Kluge & Hornick [46], 1976	North America/USA	47	M	NI	Classic	*S. schenckii*	Saprophytic	Pulmonary	Antibacterials + Potassium Iodide + Polyenes	Death
Michelson [45], 1977	North America/USA	38	M	NI	Classic	*S. schenckii*	Saprophytic	Pulmonary	Potassium Iodide + Surgical	Cure
Crout et al., [43], 1977	North America/USA	51	M	Yes	Classic	*S. schenckii*	Saprophytic	Osteoarticular	Surgical	Death
Crout et al., [43], 1977	North America/USA	54	M	Yes	Classic	*S. schenckii*	Saprophytic	Osteoarticular	Surgical + Polyenes	Sequelae
Crout et al., [43], 1977	North America/USA	25	M	NI	Classic	*S. schenckii*	Saprophytic	Osteoarticular	Surgical + Polyenes	Clinical improvement
Beardmore [44], 1979	Oceania/Australia	41	M	Yes	Classic	*S. schenckii*	Saprophytic	Disseminated Cutaneous	Potassium Iodide + Azoles + Other Antifungals + Polyenes	Cure
Matthews et al. [42], 1982	Africa/South Africa	56	F	Yes	Classic	*S. schenckii*	Saprophytic	Systemic	Potassium Iodide + Polyenes	Cure
Shelley & Sica [41], 1983	North America/USA	39	M	Yes	Classic	*S. schenckii*	Saprophytic	Systemic	Antibacterials + Potassium Iodide + Polyenes + Other Antifungals	Cure
Friedman & Doyle [40], 1983	North America/USA	71	M	Yes	Classic	*S. schenckii*	Saprophytic	Mucosal	Polyenes	Clinical improvement
Friedman & Doyle [40], 1983	North America/USA	54	M	Yes	Classic	*S. schenckii*	Saprophytic	Osteoarticular	Polyenes	Clinical improvement
Friedman & Doyle [40], 1983	North America/USA	25	M	NI	Classic	*S. schenckii*	Saprophytic	Osteoarticular	Polyenes	Cure
Friedman & Doyle [40], 1983	North America/USA	52	M	NI	Classic	*S. schenckii*	Saprophytic	Osteoarticular	Surgical	Death
Pérez et al. [56], 1992	South America/Venezuela	58	M	Yes	Classic	*S. schenckii*	Zoonotic	Disseminated Cutaneous	Potassium Iodide + Azoles	Cure
Neafie & Marty [38], 1993	North America/USA	74	M	NI	Classic	*S. schenckii*	Saprophytic	Disseminated Cutaneous	Surgical	Loss to follow-up
Saaibi et al. [37], 1996	North America/USA	36	F	NI	Classic	*S. schenckii*	NI	Systemic	Azoles	Death
Clay & Anand [36],1996	North America/USA	0.25	M	NI	Classic	*S. schenckii*	NI	Mucosal	Antibacterials + Surgery	Cure
Khabie et al. [35], 2003	North America/USA	1.58	F	Yes	Culture +Serological	*S. schenckii*	Saprophytic	Mucosal	Antibacterials + Surgery + Adjuvants/Others + Polyenes	Cure
Curi et al. [34], 2003	South America/Brazil	18	M	NI	Classic	*S. schenckii*	NI	Systemic	Polyenes	Cure
Appenzeller et al. [33], 2006	South America/Brazil	47	M	Yes	Classic	*S. schenckii*	Saprophytic	Osteoarticular	Adjuvants/Others + Surgical + Azoles	Cure
Appenzeller et al. [33], 2006	South America/Brazil	35	M	Yes	Classic	*S. schenckii*	Saprophytic	Osteoarticular	Surgical + Azoles	Cure
Yap [32], 2011	Asia/Malaysia	70	F	Yes	Classic	*S. schenckii*	Saprophytic	Disseminated Cutaneous	Polyenes + Azoles	Cure
Romero-Cabello et al. [30], 2011	Central America/Mexico	36	M	Yes	Classic	*S. schenckii*	Saprophytic	Systemic	Azoles + Potassium Iodide + Polyenes	Clinical improvement
Marques De Macedo et al. [54], 2015	South America/Brazil	9	F	Yes	Classic +Serological	*S. brasiliensis*	Zoonotic	Mucosal	Potassium Iodide + Surgical	Cure
Ribeiro et al. [31], 2015	South America/Brazil	5	M	Yes	Classic	*S. schenckii*	Zoonotic	Systemic	Polyenes + Azoles	Sequelae
Sanke et al. [29], 2016	Asia/India	54	M	Yes	Classic	*S. schenckii*	Saprophytic	Disseminated Cutaneous	Antibacterials + Azoles + Potassium Iodide	Cure
Ferreira et al. [28], 2016	South America/Brazil	35	M	Yes	Classic	*S. schenckii*	NI	Osteoarticular	Azoles	Sequelae
Hessler et al. [17], 2016	North America/USA	34	M	Yes	Classical + Serological + Molecular	*S. schenckii sensu stricto*	Saprophytic	Systemic	Antibacterials + Azoles	Clinical improvement
Mialski et al. [26], 2018	South America/Brazil	46	M	Yes	Classical + Serological + Molecular	*S. brasiliensis*	NI	Systemic	Antibacterials + Polyenes + Azoles	Sequelae
Mialski et al. [26], 2018	South America/Brazil	40	M	Yes	Classical + Serological + Molecular	*S. brasiliensis*	NI	Systemic	Antibacterials + Polyenes	Sequelae
Rueda et al. [27], 2018	South America/Peru	42	M	Yes	Classic	*S. schenckii*	Saprophytic	Mucosal	Not Informed	NI
Sun et al. [26], 2018	Asia/Japan	34	F	No	Classic	*S. schenckii*	NI	Mucosal	Surgical + Azoles	Cure
Furtado et al. [26], 2019	South America/Brazil	50	M	Yes	Classic	*S. schenckii*	Zoonotic	Mucosal	Azoles	Cure
Furtado et al. [26], 2019	South America/Brazil	46	F	Yes	Classic	*S. schenckii*	Zoonotic	Mucosal	Azoles	Cure
Reinprayoon et al. [23], 2020	Asia/Thailand	42	F	Yes	Classic + Molecular	*S. schenckii sensu stricto*	Zoonotic	Mucosal	Antibacterials + Adjuvants/Others	Cure
Sendrasoa et al. [22], 2021	Africa/Madagascar	34	M	Yes	Classic + Molecular	*S. schenckii sensu stricto*	Saprophytic	Systemic	Azoles	Cure
Martínez-Herrera et al. [21], 2021	Central America/Mexico	21	M	Yes	Classic + Molecular	*S. schenckii sensu stricto*	NI	Disseminated Cutaneous	Potassium Iodide	Cure
Zhuang et al. [20], 2022	Asia/China	47	M	Yes	Classic + Molecular	*S. globosa*	Saprophytic	Disseminated Cutaneous	Antibacterials + Azoles + Potassium Iodide	Cure
Siqueira et al. [19], 2023	South America/Brazil	78	F	Yes	Classical + Serological + Molecular	*S. brasiliensis*	Zoonotic	Osteoarticular	Antibacterials + Azoles + Polyenes	Sequelae
Siqueira et al. [19], 2023	South America/Brazil	37	F	Yes	Classical + Serological + Molecular	*S. brasiliensis*	Zoonotic	Osteoarticular	Antibacterials + Adjuvants/Others + Azoles + Polyenes	Sequelae
Rakotoarisaona et al. [18], 2024	Africa/Madagascar	35	M	Yes	Classical + Serological + Molecular	*S. schenckii sensu stricto*	Saprophytic	Disseminated Cutaneous	Azoles	Cure
Texeira et al. [55], 2024	South America/Brazil	25	F	Yes	Classic	*Sporothrix* spp.	Zoonotic	Systemic	Azoles	Sequelae

Notes: USA = United States of America; “NI” = not informed. “Exposure” refers to reported risk factors such as contact with soil, plants, wood, or animal scratches/bites (especially cats). “Diagnostic” indicates the method used (classical, immunological, molecular, or combined). “Species” corresponds to the *Sporothrix* species identified. “Transmission” is described as saprophytic (environmental) or zoonotic (animal-related). “Clinical form” specifies the main site/system affected. “Treatment” includes the therapeutic interventions used. “Outcome” indicates patient evolution: Cure (complete resolution), Clinical improvement (partial response), Sequelae (residual impairment), Disease persists (ongoing disease), Death (fatal outcome), or Loss to follow-up (not reassessed).

## Appendix C

**Glossary**

**Classical diagnosis of sporotrichosis**—refers to conventional mycological methods routinely employed in clinical practice for sporotrichosis diagnosis. These include: (1) isolation and identification of *Sporothrix* spp. in fungal culture on media such as Sabouraud dextrose agar; (2) histopathological examination of clinical specimens revealing suppurative granulomas with characteristic yeast-like cells (often cigar-shaped or asteroid bodies); and (3) direct microscopic evaluation (e.g., KOH mount) to detect fungal elements in pus, exudate, or tissue samples.

**Disseminated cutaneous sporotrichosis**—refers to a clinical form of sporotrichosis characterized by multiple, polymorphic skin lesions (ulcers, nodules, verrucous plaques) distributed across non-contiguous body sites without lymphatic spread or confirmed visceral involvement. It primarily results from hematogenous dissemination of *Sporothrix* spp., often linked to immunosuppression (e.g., HIV with low CD4 counts) but also seen in immunocompetent hosts. Lesions typically affect the trunk, face, and proximal limbs with suppurative, crusted appearances lacking marked bacterial odour, distinguishing it from primary multiple inoculations; diagnosis requires mycological confirmation via culture and histopathology showing suppurative granulomas.

**Extracutaneous sporotrichosis**—refers to forms of sporotrichosis that involve anatomical sites beyond the skin, classified according to the anatomical location of the lesions and the route of infection (primary or multifocal).

**Immunocompetent host**—refers to an individual without known conditions or therapies associated with immune dysfunction at the time of or during the course of *Sporothrix* infection, including HIV/AIDS, diabetes mellitus, chronic alcoholism, malignancy, organ transplantation, corticosteroid use, or other immunosuppressive or immunomodulatory treatments. In this systematic review, the term is used operationally to distinguish cases occurring in hosts without established predisposing factors that could confound clinical presentation, disease severity, or the interpretation of sporotrichosis manifestations.

**Molecular diagnosis**—refers to molecular methods used for species-level identification of *Sporothrix* spp., as reported in the original studies. These methods typically involved PCR amplification and sequencing of specific genetic targets for taxonomic confirmation and were not necessarily applied as primary diagnostic tools in clinical practice.

**Mucosal sporotrichosis**—refers to sporotrichosis affecting mucosal tissue, including conjunctival, nasal, and oral mucosa, as documented in the original reports. This category was applied only when infection was clearly localized to mucosal tissues and did not include lesions restricted to adjacent cutaneous areas.

**Osteoarticular sporotrichosis**—refers to sporotrichosis involving joints, tendons, or bone, typically presenting as infectious arthritis, tenosynovitis, or osteomyelitis. In most cases, osteoarticular disease arises from direct inoculation or contiguous extension from adjacent cutaneous lesions, often following deep trauma to the extremities, and is usually unifocal. Due to its distinct pathogenesis, clinical course, diagnostic features, and prognosis, osteoarticular sporotrichosis was analyzed separately in this review. Only osteoarticular manifestations associated with multifocal hematogenous dissemination and/or concomitant visceral involvement were classified as systemic, whereas isolated or contiguous osteoarticular forms were categorized separately.

**Serological diagnosis of sporotrichosis**—refers to the use of serological assays as adjunctive tools to classical diagnostic methods, such as culture or histopathology. Serology was not considered a standalone diagnostic approach for sporotrichosis, but rather as supportive evidence used to aid clinical suspicion, epidemiological assessment, or patient follow-up, reflecting its application in the original reports.

***Sporothrix schenckii***—refers to isolates reported as *Sporothrix schenckii* in the original publications based exclusively on classical diagnostic methods (culture, microscopy, and/or histopathology), without molecular typing. This designation mirrors the terminology used by the original authors—particularly in studies published before the taxonomic resolution of the *Sporothrix schenckii* complex—and does not necessarily correspond to *Sporothrix schenckii sensu stricto*.

***Sporothrix schenckii sensu stricto***—refers to cases with molecular confirmation (e.g., sequencing or species-specific PCR) that unequivocally identify the isolate as *Sporothrix schenckii sensu stricto*. In this review, the term is used to distinguish molecularly con-firmed cases from those reported as *Sporothrix schenckii* based solely on phenotypic methods, in order to enhance taxonomic accuracy and transparency.

**Saprophytic source of infection**—refers to cases in which exposure to *Sporothrix* spp. is attributed to environmental contact, such as with soil, vegetation, plant material, or other organic matter. It also includes cases in which no specific source is identified, but environmental inoculation is presumed based on the clinical and epidemiological context and the absence of documented animal-related exposure. This term is applied strictly according to the information reported in the original publications.

**Systemic sporotrichosis**—refers to sporotrichosis with confirmed involvement of deep visceral organs due to hematogenous dissemination of *Sporothrix* spp., with or without concomitant cutaneous or other extracutaneous lesions. In the clinical framework presented by Ramírez-Soto [12] 2025, this category includes pulmonary, neurological, multifocal hematogenous osteoarticular disease, sepsis, and other visceral localizations that represent true disseminated infection beyond localized cutaneous, mucosal, or purely contiguous involvement. Although systemic disease lies within the broader extracutaneous spectrum, extracutaneous involvement alone does not suffice to define systemic sporotrichosis.

**Unusual sporotrichosis**—refers to a concept-driven, operational category that encompasses severe, extracutaneous, disseminated, anatomically uncommon and/or clinically unexpected manifestations of sporotrichosis in immunocompetent hosts, in the absence of well-recognized predisposing conditions (e.g., immunosuppression, corticosteroid use, alcoholism, diabetes mellitus). This nomenclature addresses a recognized gap in the literature, as such presentations—although reported worldwide—often mimic other diseases and are therefore prone to delayed or missed diagnosis.

**Zoonotic transmission of sporotrichosis**—refers to *Sporothrix* spp. infection occurring in the context of documented or strongly suspected animal-related exposure, including bites, scratches, or direct contact with infected animals. In the studies included in this review, this category was predominantly linked to cat exposure, in line with the classifications and descriptions provided by the original authors.
